# KLF1 Promotes Non‐Small Cell Lung Cancer Cell Proliferation and Invasion by Upregulating the LINC02159/DYNC1H1 Pathway

**DOI:** 10.1002/kjm2.70070

**Published:** 2025-08-12

**Authors:** Han Yang

**Affiliations:** ^1^ Department of Medical Oncology The First Affiliated Hospital of Wenzhou Medical University Wenzhou China

**Keywords:** invasion, Kruppel‐like factor 1, LINC02159, non‐small cell lung cancer, proliferation

## Abstract

Non‐small cell lung cancer (NSCLC) is a common and fatal malignancy. This study aimed to elucidate the mechanism of Kruppel‐like factor (KLF1) in NSCLC progression. Clinical samples were collected, after which the expression levels of KLF1, LINC02159, and dynein cytoplasmic 1 heavy chain 1 (DYNC1H1) in tissues and cells were initially detected, and NSCLC cell proliferation and invasion were measured when KLF1 was up‐ or downregulated. The binding relationships among KLF1, the LINC02159 promoter, DYNC1H1, and serine and arginine‐rich splicing factor 1 (SRSF1) were analyzed. The colocalization of LINC02159 and SRSF1 was verified. DYNC1H1 stability upon actinomycin D treatment was assessed. Combined experiments were designed to confirm the interaction of the LINC02159/DYNC1H1 pathway in NSCLC development. Finally, xenograft tumors were generated in nude mice to validate the mechanism involved. KLF1, LINC02159, and DYNC1H1 were upregulated in NSCLC tissues and cells. KLF1 overexpression promoted NSCLC cell proliferation and invasion, whereas KLF1 knockdown inhibited NSCLC cell proliferation and invasion. Mechanistically, KLF1 transcriptionally activated LINC02159, which could recruit the SRSF1 protein and increase DYNC1H1 mRNA stability in the cytoplasm. Combined experiments revealed that LINC02159 and DYNC1H1 overexpression could counteract the inhibitory effect of KLF1 silencing on NSCLC cell proliferation and invasion. KLF1 silencing inhibited tumor growth in vivo by downregulating the LINC02159\DYNC1H1 pathway.

## 1 Introduction

Non‐small cell lung cancer (NSCLC) is a prevalent and life‐threatening carcinoma with an increasing death rate, and NSCLC oncogenesis and development can be attributed to smoking, lung disorders, and excessive radiation [1]. Chemotherapy and targeted therapy have significantly improved patient prognosis; however, resistance has given rise to significant challenges in treating patients with metastatic NSCLC [2]. The curative and survival rates of individuals with NSCLC have slightly improved [3]. Identifying promising biomarkers for NSCLC could provide considerable clinical benefits for NSCLC diagnosis, prognosis, and customized treatment [4]. Under these circumstances, the detection of effective biomarkers is essential for preventing NSCLC proliferation and invasion.

Transcription factors are actively dysregulated in a wide range of human carcinomas and influence cell reprogramming, metabolism, growth, and invasion [5]. As a crucial transcription factor, Kruppel‐like factor 1 (KLF1) acts as an oncogene, as it is overexpressed in a majority of malignancies and is significantly involved in cell viability, proliferation, transformation, metastasis, renewal, invasion, death, and drug resistance [6]. Notably, when KLF1 is downregulated in NSCLC, cell proliferation, migration, and invasion are correspondingly inhibited, thus preventing NSCLC progression [7]. Mechanically speaking, KLFs can mediate a variety of biological processes via crosstalk with proteins, DNA, and RNA [8]. Long noncoding RNAs (lncRNAs) are attractive targets in tumor growth and detection because their expression and transcription play vital roles in cancer biological activities [9]. Our preliminary database prediction revealed that KLF1 binds to the LINC02159 promoter. As a novel lncRNA, LINC02159 is elevated in NSCLC, which is related to increased cancer cell proliferation, metastasis, invasion, and aggressiveness, ultimately reducing cell death [10]. When LINC02159 is silenced, the NSCLC cell cycle is disrupted, tumor growth is arrested, and overall survival is promoted [10]. LncRNAs play a role in neoplastic gene interactions through an important mechanism, as they bind to downstream promoters to modulate target genes [11]. Databases have predicted that serine and arginine‐rich splicing factor 1 (SRSF1) can bind to LINC02159. SRSF1 upregulation is associated with a disappointing prognosis in patients with NSCLC, and it accelerates NSCLC cell mobility and invasion by enhancing the mRNA stability of its downstream oncogene [12]. SRSF1 can bind to dynein cytoplasmic 1 heavy chain 1 (DYNC1H1). DYNC1H1 is overexpressed in NSCLC, which promotes cell proliferation, invasion, renewal, and aggressiveness [13]. Furthermore, DYNC1H1 leads to enhanced tumor metastasis and poor prognostic outcomes in patients with NSCLC [13]. Here, we discuss the possible role of KLF1 in NSCLC proliferation and invasion through the LINC02159/DYNC1H1 pathway to provide a new theoretical basis for NSCLC prevention.

## 2 Materials and Methods

### 2.1 Clinical Sample Collection

A total of 35 patients with NSCLC who underwent surgery to remove tumors were included in our research for the collection of patients' cancerous and paracarcinoma tissues. All patients were diagnosed with NSCLC through histopathological examination, and no patients received chemotherapy or radiotherapy before surgery. Once the tissue samples were separated, they were frozen in liquid nitrogen and preserved at −80°C for further use.

### 2.2 Cell Culture

Human NSCLC cell lines (NCI‐H838, A549, PC9, and SK‐MES‐1) and bronchial epithelial cells (16HBE) (all from American Type Culture Collection, ATCC, Manassas, VA, USA) were cultivated in Dulbecco's modified Eagle's medium (HyClone Company, Logan, UT, USA) containing 10% fetal bovine serum (FBS, Yeasen Biotechnology Co. Ltd., Shanghai, China) and 1% penicillin–streptomycin (Sigma‐Aldrich, Merck KGaA, Darmstadt, Germany) in a humid incubator with 5% CO_2_ at 37°C.

### 2.3 Cell Transfection

A549 and SK‐MES‐1 cells (2 × 10^5^/well) were seeded into 6‐well plates and cultivated overnight until the cell density reached 50%–70%. Small interfering (si)RNA (GenePharma Co. Ltd., Shanghai, China) and overexpression (oe) plasmids (Hanbio Biotechnology, Shanghai, China) were transfected into cells in serum‐free medium using Lipofectamine 3000 (Invitrogen, Carlsbad, CA, USA). After 6 h of transfection, the cells were cultured in complete culture medium for 30 h.

### 2.4 Cell Counting Kit‐8 (CCK‐8) Method

Cells (1 × 10^3^) were seeded into 96‐well plates, and starting the next day, the optical density value at 450 nm (OD450) was determined with CCK‐8 kits (Cat# CK04, Dojindo Corp, Mashiki‐machi, Kumamoto, Japan). The original medium was discarded, and the cells were cultivated with a mixture of 10 μL of solution and 90 μL of medium for 2 h, followed by examination of the OD450. The growth curves were subsequently plotted according to the OD450.

### 2.5 Colony Formation Assay

A total of 500 cells were seeded into 6‐well plates. When the cells were cultivated for 2 weeks, visible clones were observed. Next, the cells were treated with 4% paraformaldehyde to fix the clones for 30 min, the methanol was discarded, the cells were stained with 0.5% crystal violet for 20 min, and the cells were photographed under a microscope.

### 2.6 Transwell Assay

A chamber (8 μm, Corning Incorporated, NY, USA) was inserted into 24‐well plates and coated with Matrigel for at least 3 h to mimic the vascular basement membrane. Subsequently, 800 μL of medium consisting of 10% FBS was added to the basolateral chamber to serve as the chemical attractant. Additionally, 1 × 10^5^ cells were added to the coated apical chamber and resuspended in 200 μL of serum‐free medium. Next, the cells invading from the apical chamber to the outward chamber through the membrane were fixed with 4% paraformaldehyde for 30 min, stained with 0.5% crystal violet for 20 min, photographed, and counted.

### 2.7 Bioinformatics Analysis

KLF1 and LINC02159 expression in NSCLC was predicted through the starBase database (https://rnasysu.com/encori/) [14]. The effect of KLF1 on NSCLC prognosis was predicted through the Kaplan–Meier plotter database (https://kmplot.com/analysis/index.php?p=service&cancer=lung) [15]. Proteins that bind to LINC02159 were predicted through the catRAPID database (http://s.tartaglialab.com/page/catrapid_group) [16], the RNAInter database (http://www.rna‐society.org/rnainter/) [17], and the RBPDB database (http://rbpdb.ccbr.utoronto.ca/) [18]. In addition, mRNAs that bind to SRSF1 were predicted through the starBase database. The binding sites between KLF1 and the LINC02159 promoter were predicted through the JASPAR website (http://jaspar.genereg.net/) [19].

### 2.8 Chromatin Immunoprecipitation (ChIP) Assay

The interaction between KLF1 and the LINC02159 promoter was verified using a ChIP Assay Kit (Millipore, Billerica, MA, USA). In brief, the cells were crosslinked with 1% formaldehyde for 10 min, followed by lysis and ultrasonication to obtain chromatin fragments. Chromatin was subsequently diluted with ChIP solution and then rotated with KLF1 (MBS421048, MyBioSource LLC, San Diego, California, USA) or immunoglobulin G (IgG, ab172730, Abcam Inc., Cambridge, MA, USA) antibodies at 4°C overnight for immunoprecipitation. After decrosslinking, the chromatin fragments were purified and analyzed by quantitative polymerase chain reaction (qPCR). The primer sequences for the LINC02159 promoter are listed in Table 1.

**TABLE 1:** Primer sequence of RT‐qPCR.

Gene	Primer sequence (5′‐3′)
KLF1	F:TCCCCCTCCTTCCTGAGTTG
	R:GGTCTCGGCTATCACACCTG
LINC02159	F:TGCAGCTTTCTATGTCCCCA
	R:ACTTGGATGTGGCCTCTGTC
DYNC1H1	F:CTTCGGAGTCACGGGTTTGA
	R:CTGGCCTTCTTCTCGGTGTT
SRSF1	F:GGTTGTCTCTGGACTGCCTC
	R:ACAAACTCCACGACACCAGT
LINC02159 promoter	F:CTATGTTGCTGAGCTGTAGTG
	R:CCAGTGTCTATTGTTCCCATC
GAPDH	F:GATGCTGGCGCTGAGTACG
	R:GCTAAGCAGTTGGTGGTGC

Abbreviations: DYNC1H1, dynein cytoplasmic 1 heavy chain 1; GAPDH, glyceraldehyde‐3‐phosphate dehydrogenase; KLF1, Kruppel‐like factor 1; RT‐qPCR, reverse transcription quantitative polymerase chain reaction; SRSF1, serine and arginine rich splicing factor 1.

### 2.9 Dual‐Luciferase Reporter Gene Assay

LINC02159‐wild type (WT) and LINC02159‐mutant type (MUT) containing binding sites with KLF1 were amplified by PCR and then cloned and inserted into pGL3‐basic luciferase reporter vectors (GeneChem, Shanghai, China). Cells were seeded into 6‐well plates, cultivated to approximately 80% confluence, and then cotransfected with oe‐KLF1 or oe‐NC and recombinant reporter plasmids for 48 h according to the instructions for recombinant protein production (Invitrogen). The cells were subsequently collected, and luciferase activity was determined using luciferase reporter assay kits (Promega, Madison, WI, USA).

### 2.10 RNA Immunoprecipitation (RIP) Assay

A RIP assay was carried out with a RIP kit (Genseed, Beijing, China). Cells (1 × 10^7^) were collected and then lysed with RIP lysis buffer solution. Then, anti‐SRSF1 (ab38017, Abcam) or NC‐IgG (ab172730, Abcam) antibodies were incubated with magnetic beads at 4°C for 2 h, followed by overnight cultivation of the cell lysates and magnetic beads at 4°C. Afterward, the cells were treated with protease K buffer to harvest immunoprecipitated RNA. Additionally, LINC02159 enrichment was assessed by reverse transcription qPCR (RT‐qPCR).

### 2.11 RNA Pull‐Down Assay

Biotin‐labeled LINC02159 was transfected using RNA Labeling Mix and T7 RNA polymerase, treated with RNase‐free DNase I (Roche, Mannheim, Germany), and purified with RNeasy Mini Kits. Total RNA was heated and annealed to form a secondary structure and mixed with cytoplasmic extract in RIP buffer at room temperature for 1 h. Next, biotinylated lncRNAs were captured using streptavidin beads (Invitrogen), and the mixture was washed and eluted. The eluate was used for western blot analysis.

### 2.12 Fluorescence In Situ Hybridization (FISH) Assay Combined With Immunofluorescence Staining

The colocalization of LINC02159 and SRSF1 was analyzed in a FISH. Briefly, the cells were washed with phosphate‐buffered saline (PBS) and fixed in 4% paraformaldehyde. Labeled LINC02159 probe (RiboBio Co. Ltd., Guangzhou, Guangdong China) was mixed with prepared hybridization buffer, in which the cells were cultivated at 37°C overnight, rinsed for 15 min, and then stained with 4′,6‐diamidino‐2‐phenylindole. Next, the cells were permeabilized with 0.1% Triton X‐100, blocked with bovine serum albumin for 1 h, and incubated with an anti‐SRSF1 antibody (ab38017, Abcam) at 4°C overnight. Additionally, the cells were cultured with an Alexa Fluor 488‐conjugated fluorescence secondary antibody (ab150077, Abcam) at room temperature for 1 h before they were photographed under a fluorescence microscope (Zeiss, Oberkochen, Germany).

### 2.13 Actinomycin D (ACT‐D) Treatment

To detect the effects of LINC02159 and SRSF1 on the DYNC1H1 mRNA attenuation rate, the cells were reacted with ACT‐D (2 μg/mL, Sigma‐Aldrich, St. Louis, MO, USA), after which the cells were harvested at 4, 8, and 12 h to extract RNA. DYNC1H1 mRNA stability was analyzed by RT‐qPCR.

### 2.14 Xenograft Tumors in Nude Mice

Male BALB/c nude mice (4 weeks old, 18–20 g) (Beijing Vital River Laboratory Animal Technology Co. Ltd., Beijing, China) were raised under standard conditions. Initially, the mice were allowed to grow for 1 week before they were randomly separated into short hairpin (sh)‐KLF1 and sh‐NC groups (mice were injected with sh‐KLF1 and sh‐NC lentiviral vectors (GeneChem), respectively). A549 cells were seeded into 6‐well plates, infected with lentivirus at a multiplicity of infection of 50, and screened with 2 μg/mL puromycin. Afterward, A549 cells (1 × 10^6^) were injected under the skin of the mice with 0.2 mL of phosphate‐buffered saline. Tumor volume was measured every 5 days from the 10th day and calculated according to the following formula: width^2^ × length × 0.5. After 30 days, the mice were euthanized with an intraperitoneal injection of pentobarbital sodium (150 mg/kg). Tumors were collected, weighed, and photographed for further analysis.

### 2.15 Immunohistochemistry (IHC)

Tumor tissues were fixed in 4% paraformaldehyde overnight, dehydrated the next day, paraffin‐embedded, and sliced into 5 μm sections, which were dehydroxylated with xylene, rehydrated with graded ethanol, and treated with 3% H_2_O_2_ to quench endogenous peroxidase activity. After antigen recovery and blocking, the sections were incubated with an anti‐Ki67 antibody (ab16667, Abcam) overnight at 4°C. The next day, the sections were cleaned, incubated with a secondary antibody (ab205718, Abcam) and visualized using 3,3′‐diaminobenzidine substrate. The stained sections were photographed under a light microscope and analyzed by investigators unaware of the experimental groups.

### 2.16 RT‐qPCR

TRIzol reagent (Invitrogen) was used to extract total RNA, which was then reverse transcribed into cDNA using EntiLink first‐strand cDNA synthesis kits (ELK Biotechnology, Hamburg Germany). RT‐qPCR was performed using a StepOne Real‐Time PCR system (Thermo Fisher Scientific, Waltham, MA, USA). Glyceraldehyde‐3‐phosphate dehydrogenase served as the internal reference. The 2^–ΔΔCT^ method was used to measure relative expression [20]. The primer sequences are listed in Table 1.

### 2.17 Western Blot Analysis

Total protein was extracted with radioimmunoprecipitation assay lysis buffer (Aspen Biotechnology, Wuhan, Hubei, China), and the concentration of total protein was analyzed using bicinchoninic acid kits. Proteins were subsequently separated by sodium dodecyl sulfate–polyacrylamide gel electrophoresis and then transferred onto polyvinylidene fluoride membranes, which were then incubated with primary antibodies (PA5‐86441, 1:1000, Thermo Fisher, Waltham, MA, USA), DYNC1H1 (ab245554, 1:2000, Abcam), SRSF1 (ab38017, 1:1000, Abcam), and β‐actin (ab5694, 1:1000, Abcam) at 4°C overnight. After that, the membranes were incubated with an IgG secondary antibody (ab205718, 1:2000, Abcam) at room temperature for 1 h. Protein bands were observed with an enhanced chemiluminescence kit, with β‐actin as an internal reference.

### 2.18 Statistical Analysis

SPSS 21.0 (IBM SPSS Statistics, Chicago, IL, USA) was used for data analysis, and GraphPad Prism 8.0 software (GraphPad Software Inc., San Diego, CA, USA) was used for graphing. The results are presented as the means ± standard deviations. All the data were inspected for a normal distribution and homogeneity of variance. Comparisons between two groups were analyzed with a *t* test, and comparisons among multiple groups were analyzed with one‐ or two‐way analysis of variance (ANOVA). Tukey's multiple comparisons test was used for post hoc tests. The *p* values were obtained by two‐tailed tests, with *p* < 0.01 indicating extremely significant differences.

## 3 Results

### 3.1 KLF1 is Overexpressed in NSCLC and Enhances NSCLC Cell Proliferation and Invasion

**FIGURE 1:**
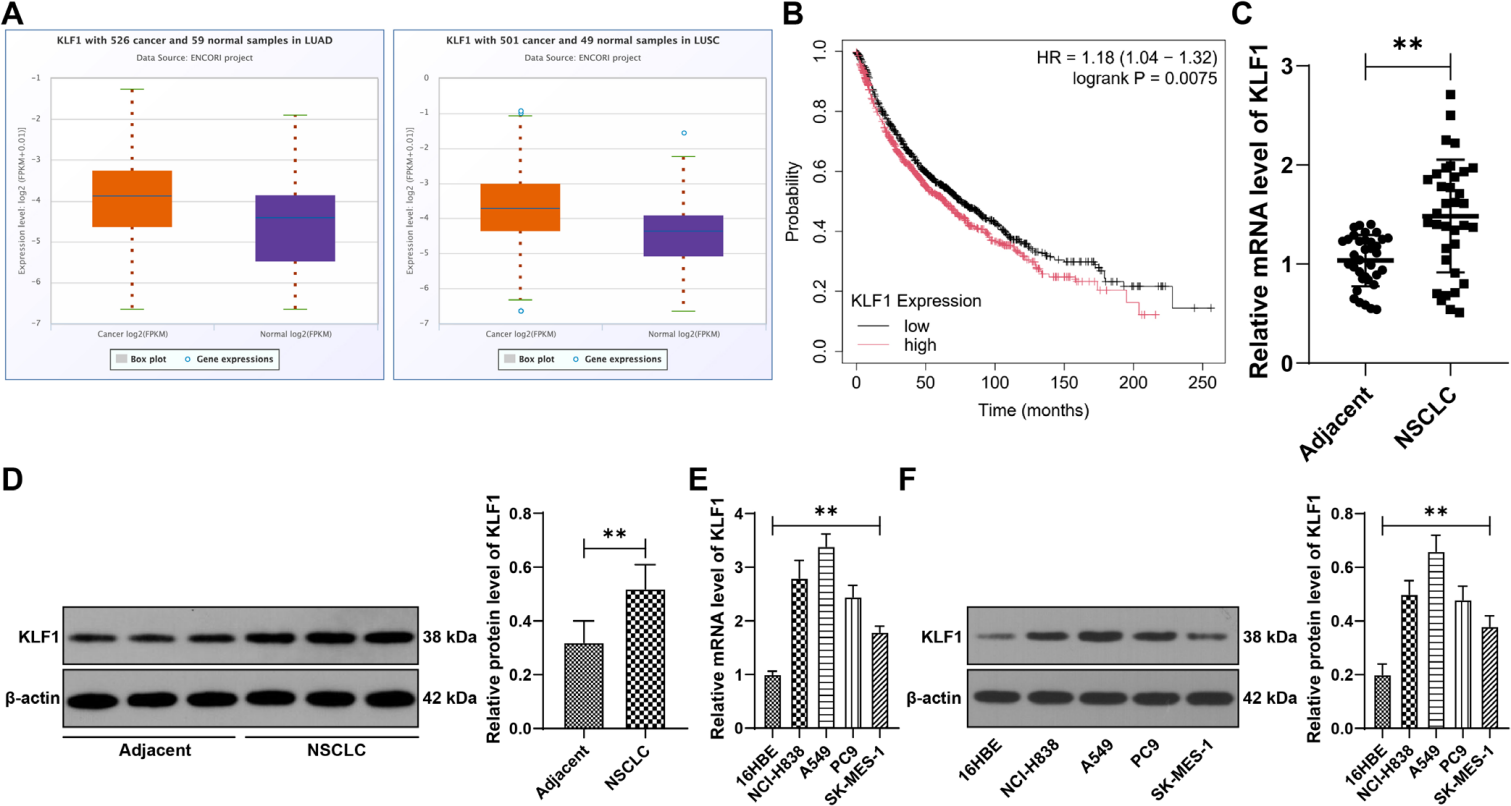
KLF1 is overexpressed in NSCLC. (A) KLF1 expression in NSCLC was predicted through the starBase database. (B) The relationship between KLF1 expression and NSCLC patient prognosis was predicted through the Kaplan–Meier plotter database. (C–F) KLF1 expression was detected by RT‐qPCR and western blot analysis. *N* = 35. All steps were repeated three times. The data are presented as the means ± standard deviations. A *t* test was used to analyze the data in panels C and D, and one‐way ANOVA was used to analyze the data in panels E and F. **p < 0.01.

**FIGURE 2:**
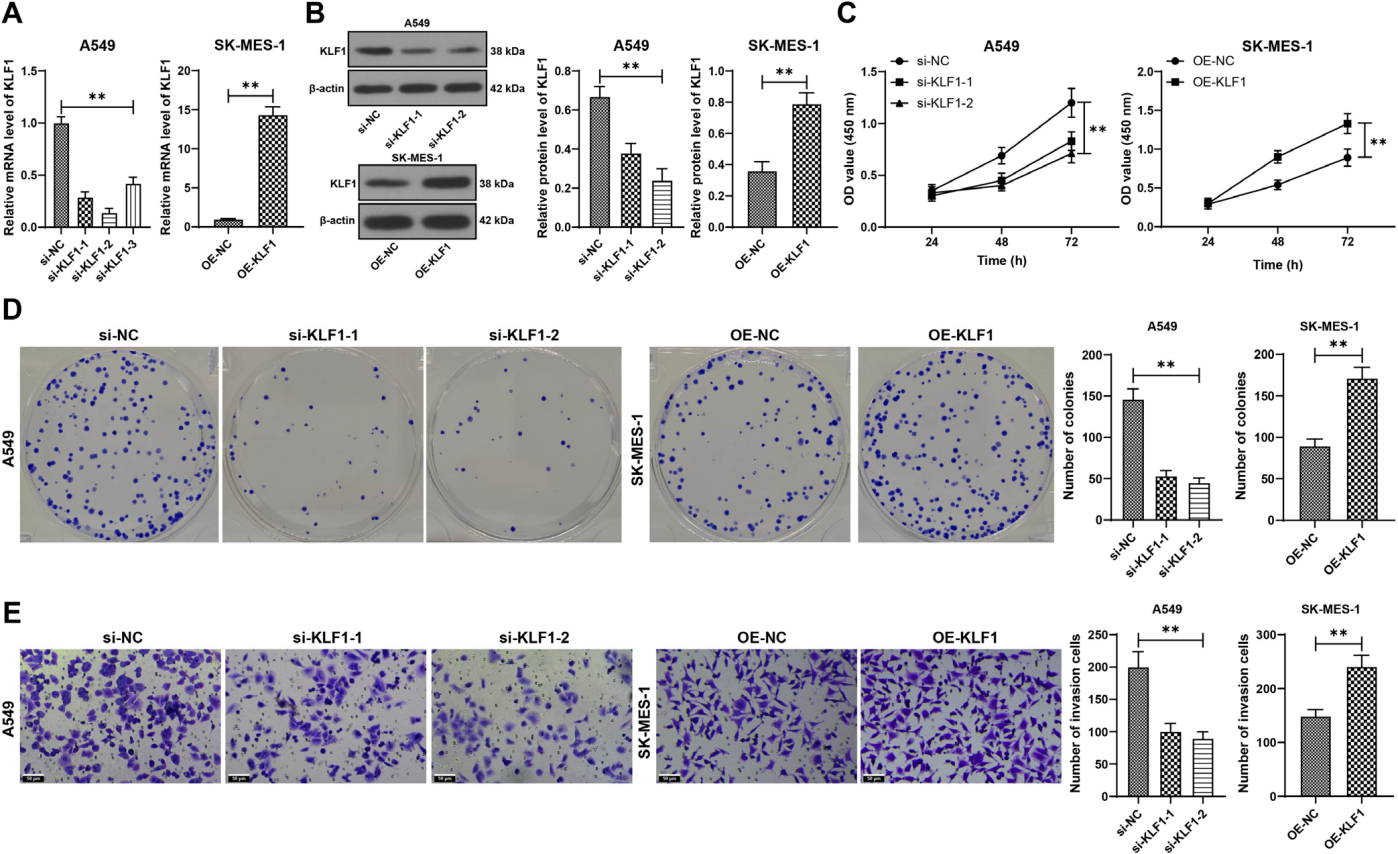
KLF1 enhances NSCLC cell proliferation and invasion. si‐KLF1 was transfected into A549 cells, and oe‐KLF1 was transfected into SK‐MES‐1 cells, with si‐NC or oe‐NC as a control. A and B, KLF1 expression in cells was assessed by RT‐qPCR (A) and western blot analysis (B). (C and D) CCK‐8 (C) and colony formation (D) assays were utilized to examine cell proliferation. (E) Cell invasion was determined by a Transwell assay. All steps were repeated three times. The data are presented as the means ± standard deviations. The *t* test was used to analyze the data in the right panels of A, B, D, and E; one‐way ANOVA was used to analyze the data in the left panels of A, B, D, and E; and two‐way ANOVA was used to analyze the data in panel C. Tukey's multiple comparisons test was used for post hoc tests. **p < 0.01.

According to the starBase database prediction, KLF1 was abundantly expressed in NSCLC (Figure 1A). The Kaplan–Meier plotter database predicted that patients with NSCLC with overexpressed KLF1 had poor prognoses (*p* < 0.01, Figure 1B). NSCLC tissues and cells presented increased KLF1 expression (*p* < 0.01, Figure 1C–F). Because A549 cells had relatively high KLF1 expression, whereas SK‐MES‐1 cells had low KLF1 expression, functional verification was performed on these two cell lines. In A549 cells, si‐KLF1 was used to silence KLF1 expression (*p* < 0.01, Figure 2A,B), and 2 siRNAs with high transfection efficiency were selected for subsequent experiments. Moreover, oe‐KLF1 was transfected into SK‐MES‐1 cells (*p* < 0.01, Figure 2A,B). Our results revealed that KLF1 silencing led to decreased A549 cell proliferation, colony formation, and invasion, whereas overexpression of KLF1 led to improved cell proliferation and invasion (*p* < 0.01, Figure 2C–E), suggesting that KLF1 is overexpressed in NSCLC and that it enhances NSCLC cell proliferation and invasion.

### 3.2 KLF1 Could Activate LINC02159 via Transcriptional Regulation

**FIGURE 3:**
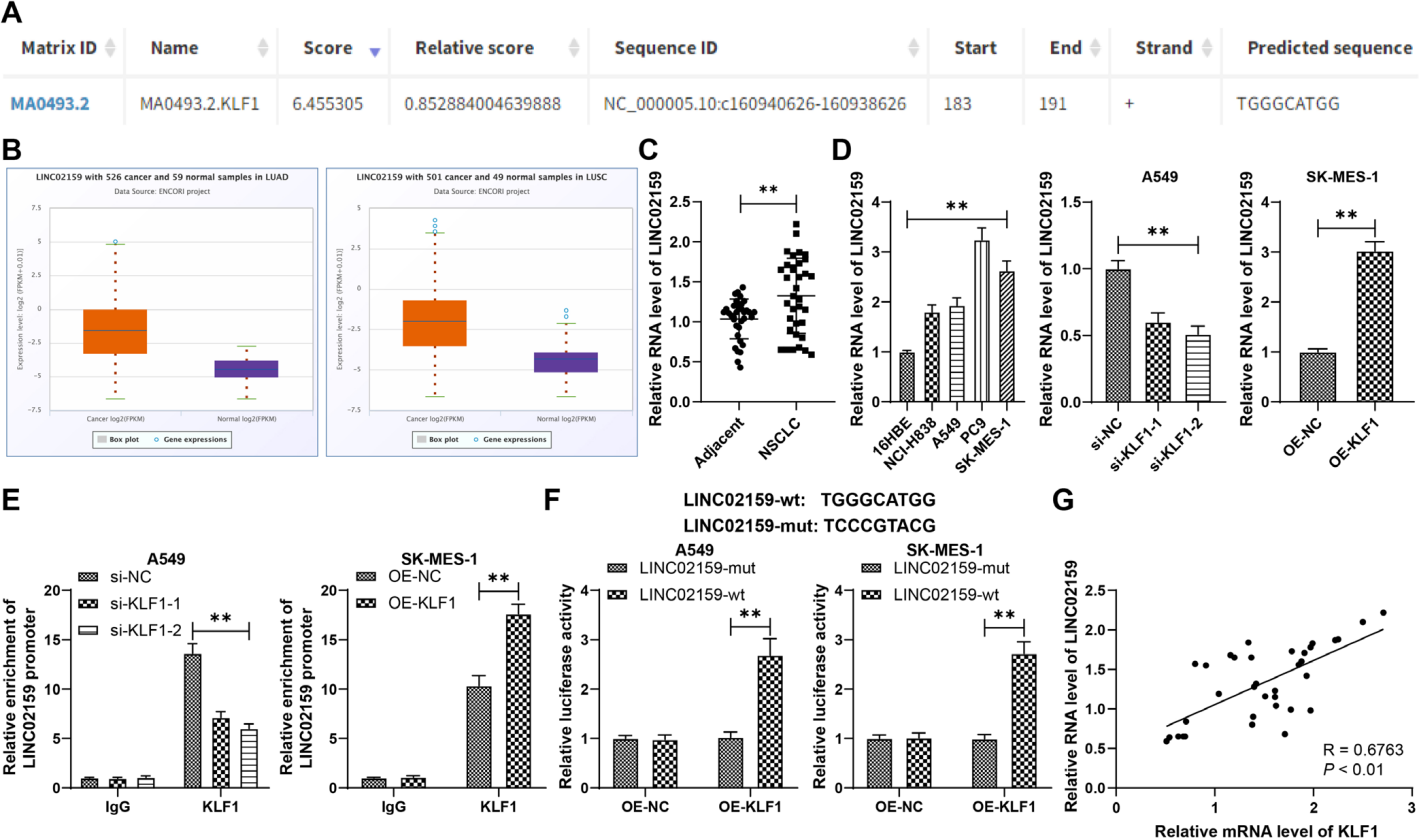
KLF1 could activate LINC02159 via transcriptional regulation. (A) Binding sites between KLF1 and the LINC02159 promoter were predicted through a database. (B) LINC02159 expression in NSCLC was predicted through the starBase database. (C and D) LINC02159 expression in NSCLC tissues and cells was determined by RT‐qPCR. (E and F) The binding relationship between KLF1 and the LINC02159 promoter was validated via ChIP (E) and dual‐luciferase reporter gene (F) assays. (G) Relationships between KLF1 mRNA and LINC02159 expression in NSCLC tissues were analyzed by Person correlation analysis. *N* = 35. All steps were repeated three times. The data are presented as the means ± standard deviations. The *t* test was employed to analyze the data in panel C and the right panel of D, one‐way ANOVA was used to analyze the data in panel D, and two‐way ANOVA was used to analyze the data in panels E and F. Tukey's multiple comparisons test was applied for the post hoc test. **p < 0.01.

As a transcription factor, KLF1 can regulate its downstream gene expression. KLF1 can bind to LINC02159 (Figure 3A), and LINC02159 can accelerate NSCLC progression through the ALYREF/YAP1 pathway [10]. The starBase database revealed that LINC02159 was upregulated in NSCLC (Figure 3B). These results indicated that LINC02159 might be a possible downstream mechanism of KLF1. LINC02159 expression was verified, and the results revealed that compared with adjacent tissues or 16HBE cells, NSCLC cancerous tissues and cells presented increased LINC02159 expression (*p* < 0.01, Figure 3C,D). Compared with the si‐NC group, the si‐KLF1 group presented lower LINC02159 expression, and the oe‐KLF1 group presented higher LINC02159 expression than the oe‐NC group did (*p* < 0.01, Figure 3C,D). According to the binding sites predicted in Figure 3A and the results of the ChIP assay, KLF1 was enriched in the LINC02159 promoter region, and silencing KLF1 was linked to decreased enrichment, whereas overexpression of KLF1 led to increased enrichment (*p* < 0.01, Figure 3E). The binding relationship between KLF1 and LINC02159 was further validated in a dual‐luciferase reporter gene assay (*p* < 0.01, Figure 3F). KLF1 mRNA and LINC02159 expression levels were positively related in NSCLC tissues (*p* < 0.01, Figure 3G). These findings indicated that KLF1 could activate LINC02159 via transcriptional regulation.

### 3.3 KLF1 Promotes NSCLC Cell Proliferation and Invasion by Activating LINC02159 Expression

**FIGURE 4:**
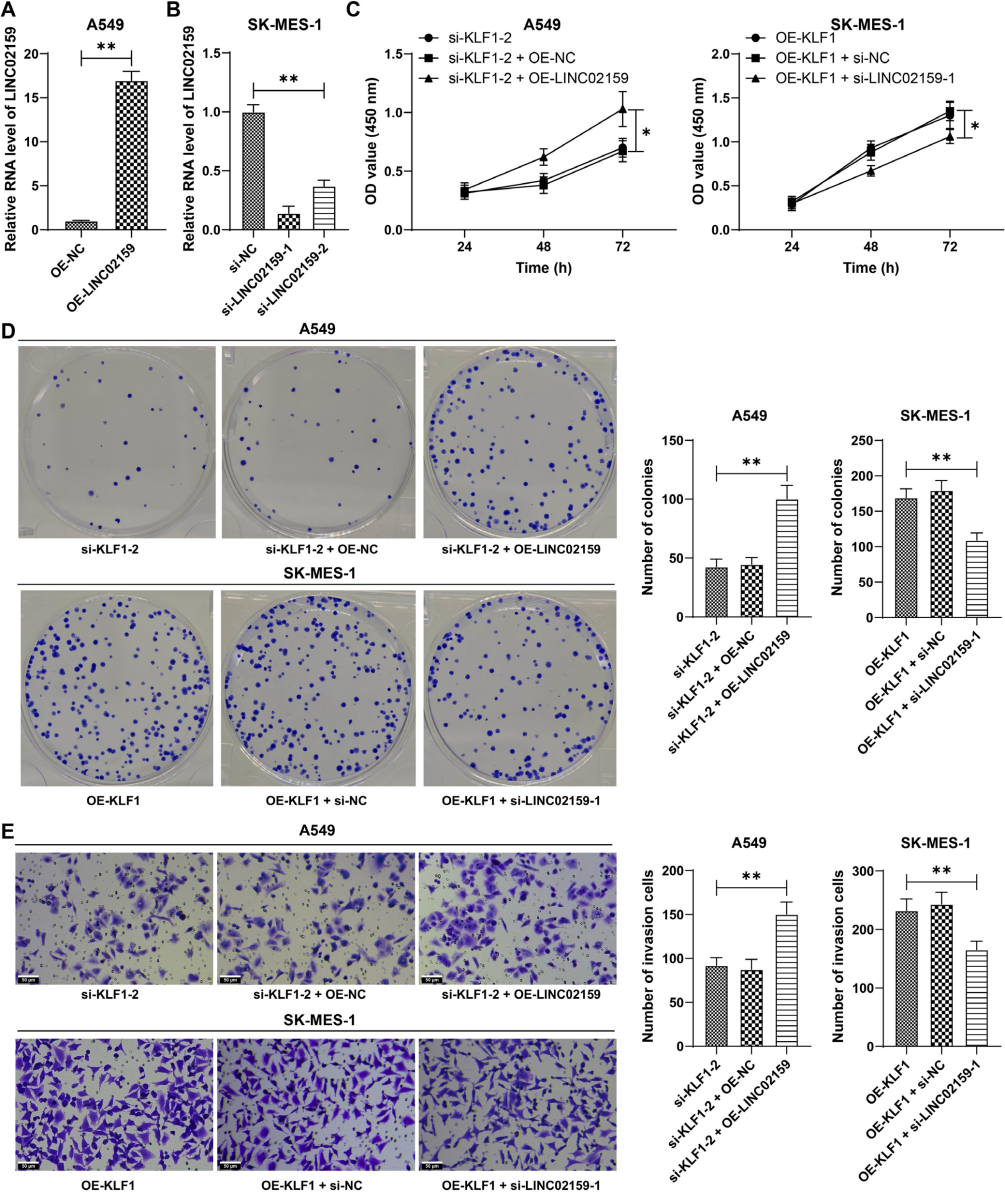
KLF1 promotes NSCLC cell proliferation and invasion by activating LINC02159 expression. oe‐LINC02159 was transfected into A549 cells, and si‐LINC02159 was transfected into SK‐MES‐1 cells, with oe‐NC or si‐NC as a control. (A and B) LINC02159 expression in cells was examined by RT‐qPCR. (C and D) Cell proliferation was assessed by CCK‐8 (C) and colony formation (D) assays. (E) Cell invasion was measured by a Transwell assay. Independent experiments were repeated three times. The data are presented as the means ± standard deviations. A *t* test was used to analyze the data in panel A; one‐way ANOVA was used to analyze the data in panels B, D, and E; and two‐way ANOVA was used to analyze the data in panel C. Tukey's multiple comparisons test was used for post hoc tests. *p < 0.05, **p < 0.01.

Combined experiments were conducted in A549 cells, as LINC02159 was overexpressed and KLF1 was silenced (*p* < 0.01, Figure 4A). Similarly, in SK‐MES‐1 cells, LINC02159 expression was downregulated, and KLF1 expression was upregulated (*p* < 0.01, Figure 4B). When LINC02159 was upregulated, cell proliferation was increased, and LINC02159 silencing increased cell proliferation (*p* < 0.01, Figure 4C,D). In addition, cell invasion was promoted upon LINC02159 overexpression but impaired upon LINC02159 silencing (*p* < 0.01, Figure 4E). These results indicated that KLF1 promoted NSCLC cell proliferation and invasion by activating LINC02159 expression.

### 3.4 LINC02159 Recruits the SRSF1 Protein to Increase DYNC1H1 mRNA Stability in the Cytoplasm

**FIGURE 5:**
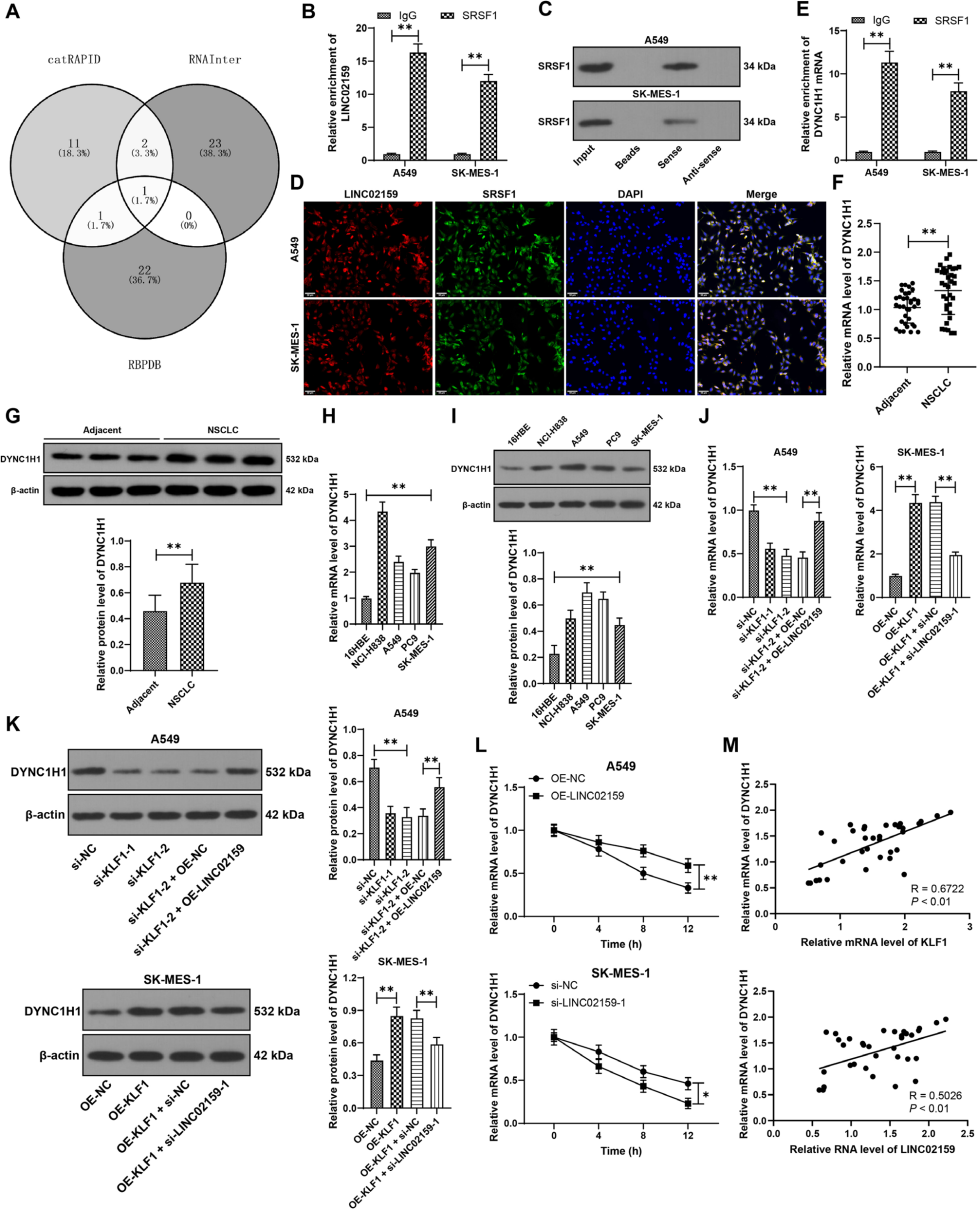
LINC02159 recruits the SRSF1 protein to increase DYNC1H1 mRNA stability in the cytoplasm. (A) Proteins that bind to LINC02159 were predicted through databases, and the intersection was captured. (B and C) The binding relationship between LINC02159 and SRSF1 was verified by RIP and RNA pull‐down assays. (D) Colocalization of LINC02159 and SRSF1 in cells was analyzed by FISH combined with immunofluorescence staining. (E) The binding relationship between SRSF1 and DYNC1H1 mRNA was assessed in a RIP assay. (F–K) DYNC1H1 expression in tissues and cells was detected by RT‐qPCR and western blot analysis. (L) DYNC1H1 mRNA stability after ACT‐D treatment was determined by RT‐qPCR. (M) Relationships between KLF1 mRNA expression, LINC02159 expression and DYNC1H1 mRNA expression in NSCLC tissues were analyzed by Pearson correlation analysis. *N* = 35. Independent experiments were repeated three times. The data are presented as the means ± standard deviations. A *t* test was used to analyze the data in panels F and G; one‐way ANOVA was used to analyze the data in panels H, I and K; and two‐way ANOVA was used to analyze the data in panels B, E, and L. Tukey's multiple comparisons test was used for post hoc tests. *p < 0.05, **p < 0.01.

LINC02159 can recruit the PTBP1 protein, thereby increasing LATS2 mRNA stability in the cytoplasm [21]. Information available on databases, including catRAPID, RBPDP, and RNAInter, revealed that SRSF1 bound to LINC02159 (Figure 5A). The results of the RIP assay demonstrated that LINC02159 was significantly enriched in the SRSF1 coprecipitation group (*p* < 0.01, Figure 5B). An RNA pull‐down assay revealed that LINC02159 downregulated the SRSF1 protein (Figure 5C). The colocalization and binding relationship between LINC02159 and SRSF1 in NSCLC cells were measured by FISH and immunofluorescence staining, which revealed strong colocalization between LINC02159 and the SRSF1 protein in cells, especially in the cytoplasm (Figure 5D). The starBase database predicted that SRSF1 bound to DYNC1H1. DYNC1H1 has been reported to be highly expressed in NSCLC [13]. The SRSF1 antibody strengthened DYNC1H1 mRNA enrichment (*p* < 0.01, Figure 5E), indicating the binding relationship between SRSF1 and DYNC1H1. In addition, DYNC1H1 expression was upregulated in NSCLC tissues and cells (*p* < 0.01, Figure 5F–I). Silencing KLF1 reduced DYNC1H1, which was counteracted by LINC02159 overexpression, whereas KLF1 overexpression increased DYNC1H1 expression, and LINC02159 inactivation reduced DYNC1H1 expression (*p* < 0.01, Figure 5J”K). LINC02159 overexpression improved DYNC1H1 mRNA stability, and LINC02159 knockdown reversed this effect (*p* < 0.01, Figure 5L). KLF1 mRNA, LINC02159 expression, and DYNC1H1 mRNA were positively related in NSCLC tissues (*p* < 0.01, Figure 5M). The above data illustrate that LINC02159 recruits the SRSF1 protein to increase DYNC1H1 mRNA stability and DYNC1H1 expression.

### 3.5 KLF1 Accelerates NSCLC Cell Proliferation and Invasion by Activating the LINC02159/DYNC1H1 Pathway

**FIGURE 6:**
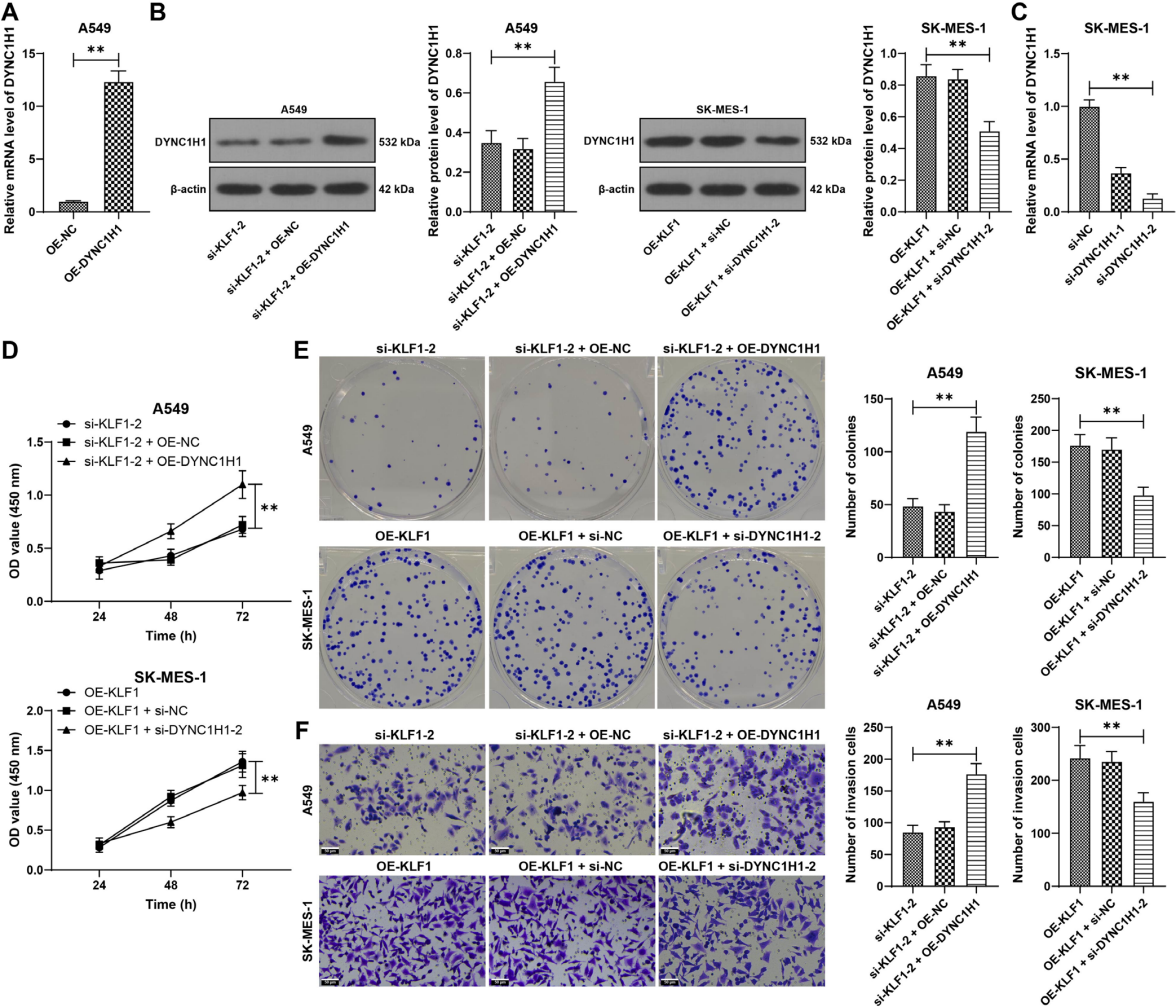
KLF1 accelerates NSCLC cell proliferation and invasion by activating the LINC02159/DYNC1H1 pathway. oe‐DYNC1H1 was transfected into A549 cells, and si‐DYNC1H1 was transfected into SK‐MES‐1 cells, with oe‐NC or si‐NC as a control. (A–C) DYNC1H1 expression in cells was examined by RT‐qPCR and western blot analysis. (D and E) Cell proliferation was assessed by CCK‐8 (D) and colony formation (E) assays. (F) Cell invasion was measured in a Transwell assay. Independent experiments were repeated three times. The data are presented as the means ± standard deviations. The *t* test was employed to analyze the data in panel A; one‐way ANOVA was used to analyze the data in panels B, C, E, and F; and two‐way ANOVA was used to analyze the data in panel D. Tukey's multiple comparisons test was applied for the post hoc test. **p < 0.01.

Combined experiments were conducted in A549 cells, as DYNC1H1 was upregulated and KLF1 was downregulated (*p* < 0.01, Figure 6A,B). Similarly, combined experiments were conducted in SK‐MES‐1 cells, as DYNC1H1 was inactivated and KLF1 was activated (*p* < 0.01, Figure 6B,C). Overexpressing DYNC1H1 facilitated NSCLC cell proliferation and invasion, which was reversed by DYNC1H1 knockdown (*p* < 0.01, Figure 6D–F). Overall, KLF1 accelerated NSCLC cell proliferation and invasion by activating the LINC02159/DYNC1H1 pathway.

### 3.6 KLF1 Silencing Downregulates the LINC02159/DYNC1H1 Pathway to Inhibit Tumor Growth In Vivo

**FIGURE 7:**
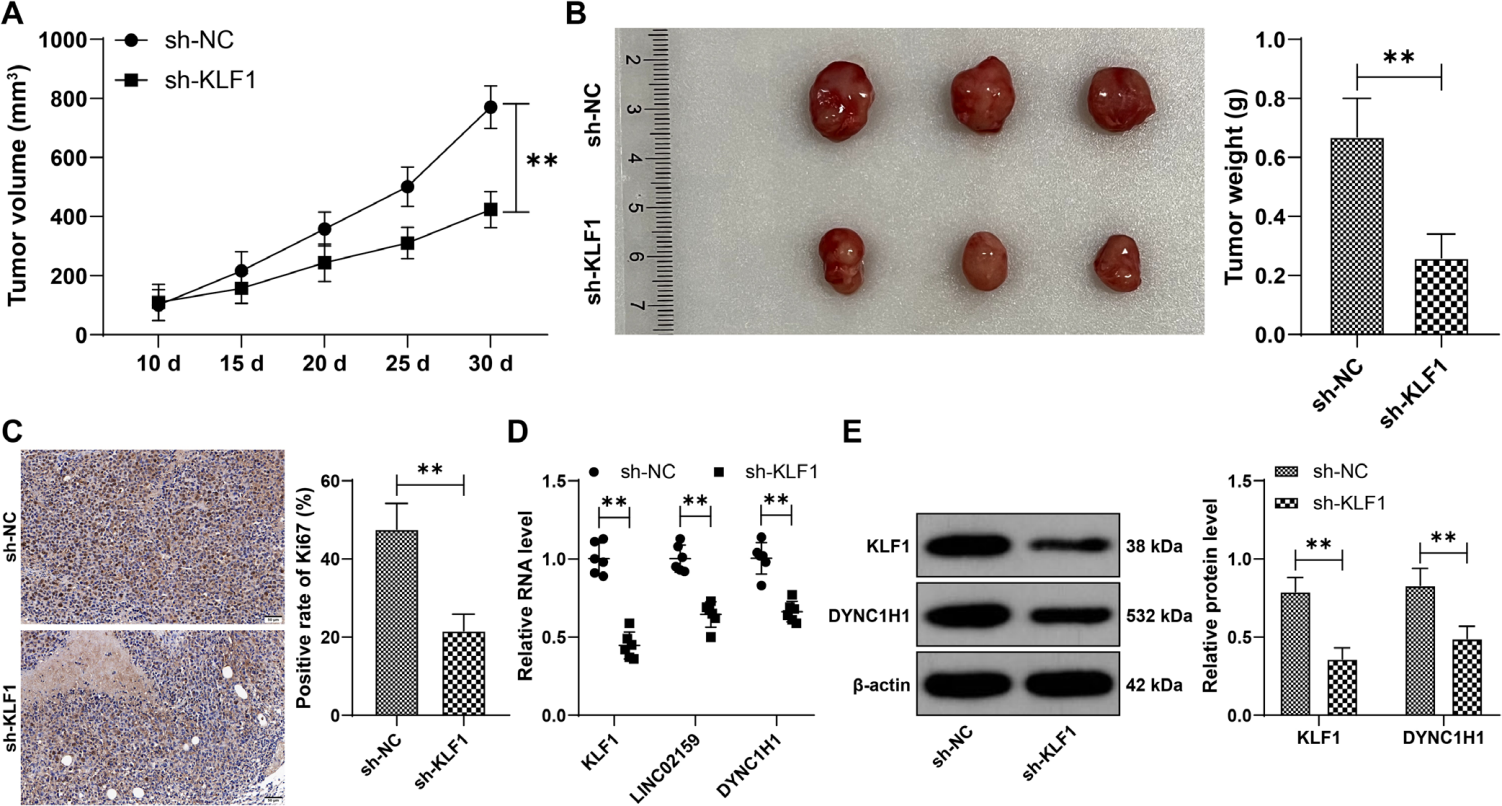
KLF1 silencing downregulates the LINC02159/DYNC1H1 pathway to inhibit tumor growth in vivo. A549 cells with different infection treatments were utilized to establish xenograft tumors in nude mice. (A) The tumor volume was recorded every 5 days. (B) Tumor weights were measured. (C) Ki67‐positive rates were detected by IHC. (D and E) The expression levels of KLF1, LINC02159 and DYNC1H1 were verified by RT‐qPCR and western blot analysis. *N* = 6. The data are presented as the means ± standard deviations. A *t* test was used to analyze the data in panels B and C, and two‐way ANOVA was used to analyze the data in panels A, D, and E. Tukey's multiple comparisons test was used for post hoc tests. **p < 0.01.

**FIGURE 8:**
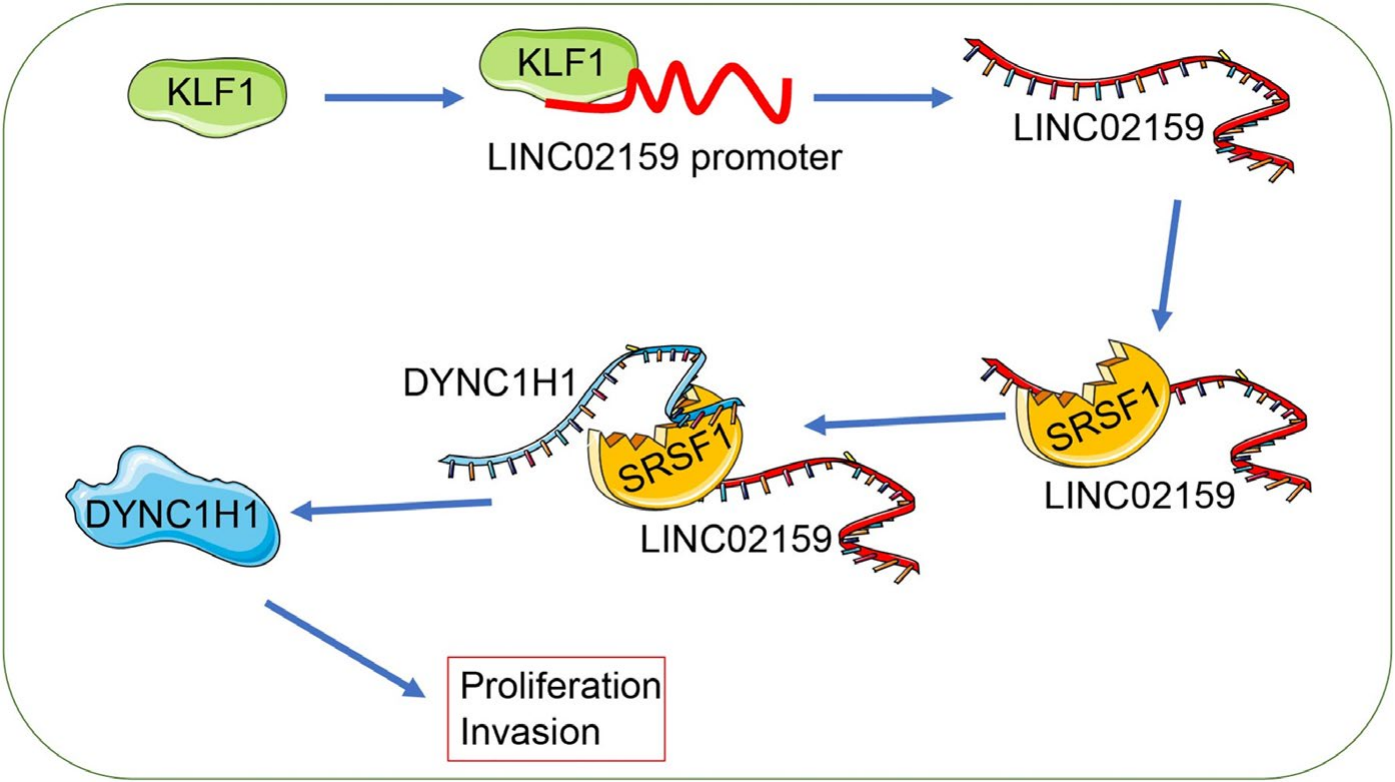
Mechanism by which KLF1 promotes NSCLC cell proliferation and invasion. KLF1 binds to the LINC02159 promoter to upregulate LINC02159 expression, and LINC02159 recruits the SRSF1 protein and enhances DYNC1H1 mRNA stability to promote DYNC1H1 expression to accelerate NSCLC cell proliferation and invasion.

A549 cells were utilized to establish xenograft tumors in nude mice. KLF1 silencing led to decreased tumor volume and weight (*p* < 0.01, Figure 7A,B) and decreased the Ki67‐positive rate of proliferation (*p* < 0.01, Figure 7C). Compared with those in the sh‐NC group, the levels of KLF1, LINC02159, and DYNC1H1 were lower in the sh‐KLF1 group (*p* < 0.01, Figure 7D,E). Overall, KLF1 silencing downregulated the LINC02159/DYNC1H1 pathway to inhibit tumor growth in vivo (Figure 8).

## 4 Discussion

As the most predominant cancer, NSCLC is linked to impaired quality of life, a decreased overall survival rate, and increased probability of recurrence; therefore, novel strategies for treating NSCLC are needed [22]. Transcription factors participate in gene regulation and cell biological activities in NSCLC [23]. As a group of transcription factors, KLFs are considered detrimental factors in different carcinomas [24]. In the present study, we investigated the interaction between KLF1 and NSCLC cell proliferation and invasion with the involvement of the LINC02159/DYNC1H1 pathway.

The first principal finding from our study was that KLF1 was strongly expressed in NSCLC and that KLF1 overexpression enhanced cell proliferation and invasion. KLFs are proactive cytokines in NSCLC progression [25]. KLF1 is highly expressed in lung cancer cells, and downregulation of KLF1 inhibits the migration of NSCLC cells and induces apoptosis [7]. Similarly, KLF1 significantly increased cell proliferation, transformation, and epithelial–mesenchymal transition in gastric cancer [26]. Overall, strongly expressed KLF1 could be recognized as a risk manifestation in human cancers. Increasing evidence has shown that KLF1, an essential coordinating factor, aggravates a variety of malignancies by enhancing cell migration, invasion, and resistance to therapies through the orchestration of its downstream gene translation and expression [27, 28]. Therefore, we explored the possible targets downstream of KLF1 through the JASPAR database, from which LINC02159 was captured. Notably, LINC02159 functions as a dangerous factor and a hub cytokine in hepatocellular carcinoma development and the prognosis network [29], inspiring us to further elucidate the mechanism of LINC02159 in NSCLC.

The results of our combined experiments revealed that KLF1 promoted NSCLC cell proliferation and invasion by activating LINC02159 expression via transcriptional regulation. LncRNAs participate in many cell biological behaviors and serve as oncogenes or tumor suppressors, such as in NSCLC [30]. LINC02159 is differentially expressed in a substantial number of carcinomas and is necessarily involved in cancer detection, classification, growth, and prognosis [31]. Importantly, when LINC02159 was silenced in NSCLC, cell growth and invasion were blocked, and cancer cell death was promoted [10]. Furthermore, our experiments revealed strong colocalization between LINC02159 and the SRSF1 protein in the cytoplasm. As a classic splicing controller, SRSF1 is dysregulated in the progression of multiple types of tumors through a molecular network that is regulated by upstream factors via binding sites, thereby affecting mRNA programming and biological behaviors [32]. Terroba et al. reported that SRSF1 binds to its downstream gene and promotes the stability of its mRNA to increase gene expression, thereby accelerating NSCLC cell proliferation and mobility [12], which is consistent with our finding that SRSF1 enhances DYNC1H1 mRNA stability to upregulate DYNC1H1 expression. DYNC1H1 serves as an intriguing biomarker in different kinds of cancers, as the variance in its mRNA stability could alter cancer cell repair, metabolism, and invasion [33]. High DYNC1H1 expression in lung cancer tissues was significantly associated with clinical tumor stage, distal metastasis, and poor prognosis [13]. Our experimental results further revealed that upregulation of DYNC1H1 resulted in a significant increase in the number of proliferating and invading NSCLC cells, whereas downregulation of DYNC1H1 resulted in the opposite trend, suggesting that DYNC1H1 might be an oncogene in the tumor microenvironment. This mechanism was validated by in vitro experiments.

## 5 Conclusion

Overall, KLF1 transcriptionally activated LINC02159, which could recruit SRSF1 protein and increase DYNC1H1 mRNA stability in the cytoplasm to facilitate DYNC1H1 expression, thereby increasing NSCLC cell proliferation and invasion. These results are useful for designing possible treatments for NSCLC. Nevertheless, several limitations remain. Considering that our study is only a preliminary exploration, our single mechanism did not reveal whether KLF1 could regulate the epithelial–mesenchymal transition or ferroptosis of NSCLC cells. In addition, other downstream miRNAs of LINC02159 should be investigated. Moreover, in this study, we discuss only the involvement of SRSF1 in NSCLC; whether RBM6 and LIN28A are involved in this mechanism remains to be further explored. Thus, in our future studies, the interaction between KLF1 and epithelial–mesenchymal transition or ferroptosis will be studied. Clinical samples will be included if permitted, and a deep understanding of the LINC02159 downstream mechanism will be sought to provide a novel theoretical basis for NSCLC mitigation.

## Ethics Statement

This research was granted and monitored by the ethics committee of our hospital. All the subjects signed informed consent forms. Each step was rigorously performed according to the *Declaration of Helsinki*. The protocol was also guided by the Institutional Animal Care and Use Committee of our hospital and *Guidelines for the Care and Use of Laboratory Animals* proposed by the National Institutes of Health. Significant efforts were made to reduce the number of animals used and their suffering.

## Conflicts of Interest

The author declares no conflicts of interest.

## Data Availability

The data that support the findings of this study are available from the corresponding author upon reasonable request.
